# Exercise-induced metabolic remodeling in type 2 diabetes mellitus: insights from metabolomics

**DOI:** 10.3389/fendo.2026.1939488

**Published:** 2026-09-04

**Authors:** Huan Xiang, Jing Miao, Jinfang Cheng, Jiheng Yuan, Hongyu Gong, Yumei Han, Linlin Gao

**Affiliations:** 1 School of Physical Education, Shanxi University, Taiyuan, China; 2 Department of Cardiovascular Medicine, Shanxi Bethune Hospital, Shanxi Academy of Medical Sciences, Tongji Shanxi Hospital, Third Hospital of Shanxi Medical University, Taiyuan, China; 3 Department of Endocrinology, Shanxi Bethune Hospital, Shanxi Academy of Medical Sciences, Tongji Shanxi Hospital, Third Hospital of Shanxi Medical University, Taiyuan, China

**Keywords:** exercise intervention, insulin resistance, metabolic remodeling, metabolomics, precision exercise medicine, type 2 diabetes mellitus

## Abstract

Type 2 diabetes mellitus (T2DM) is one of the most pressing global public health challenges. Exercise intervention, as a cost-effective and safe non-pharmacological strategy, exerts its metabolic benefits through coordinated multi-organ and multi-target regulation. Metabolomics technologies provide powerful tools for dissecting the molecular mechanisms underlying exercise intervention in T2DM. This narrative review summarizes current evidence on metabolic dysregulation in T2DM and the metabolic mechanisms underlying exercise intervention, with particular emphasis on insights provided by metabolomics. Furthermore, it elaborates the mechanisms by which exercise intervention systemically reverses T2DM-associated metabolic abnormalities: restoring tricarboxylic acid cycle flux to alleviate glycolytic stress; accelerating branched-chain amino acid oxidative catabolism to rectify aromatic amino acid metabolic disturbances; and promoting fatty acid oxidation while clearing lipotoxic products. In addition, this review highlights the clinical potential of metabolomics for predicting exercise responsiveness, dynamically evaluating intervention outcomes and safety, thereby providing a potential basis for more individualized exercise intervention strategies in T2DM.

## 1 Introduction

Driven by global population aging and lifestyle changes, the incidence of type 2 diabetes mellitus (T2DM) and its associated complications has increased steadily, making T2DM one of the most pressing global public health challenges (1). According to the International Diabetes Federation (IDF) Diabetes Atlas (2), an estimated 589 million adults aged 20–79 years were living with diabetes worldwide in 2024, corresponding to a prevalence rate of 11.1%—approximately one in every nine adults. Exercise intervention, as a cost-effective and safe non-pharmacological strategy, has been widely recommended for the prevention and management of diabetes (3). Regular physical activity can improve glycemic control, enhance insulin sensitivity, regulate lipid metabolism, and reduce the risk of cardiovascular and other complications.

However, the molecular mechanisms underlying the metabolic benefits of exercise are highly complex and involve coordinated crosstalk among multiple organs, including skeletal muscle, the liver, adipose tissue, and the gut (4). It is difficult to fully elucidate such a multi-organ, multi-target regulatory network using conventional approaches. Recent advances in metabolomics have provided powerful tools for addressing this challenge (5). By enabling the high-throughput profiling of dynamic changes in small-molecule metabolites within biological samples, metabolomics can systematically delineate the metabolic remodeling landscape under exercise intervention, identify key biomarkers, and reveal novel regulatory pathways (6).

## 2 Methods

A narrative literature search was conducted to identify relevant studies on metabolic alterations in type 2 diabetes mellitus (T2DM), metabolic responses to exercise, and the application of metabolomics in exercise intervention. PubMed, Web of Science, Scopus, and Google Scholar were searched from database inception to August 2026. The search strategy combined terms related to T2DM (“type 2 diabetes” OR “type 2 diabetes mellitus”), exercise (“exercise” OR “physical activity” OR “exercise intervention”), and metabolomics (“metabolomics” OR “metabolomic profiling” OR “metabolic flux analysis”). Additional relevant studies were identified by screening the reference lists of key articles. Studies were considered based on their relevance to metabolic dysregulation in T2DM, exercise-induced metabolic adaptations, metabolomics technologies, and the potential clinical translation of metabolic biomarkers.

## 3 Core metabolic disturbances in T2DM

### 3.1 Glucose metabolism and energy metabolism

The hallmark pathological features of T2DM are insulin resistance (IR) and pancreatic β-cell dysfunction, which together lead to systemic disturbances in glucose metabolism. Under physiological conditions, efficient glucose uptake by peripheral tissues (primarily skeletal muscle) (7), fine-tuned regulation of hepatic gluconeogenesis and glycogenolysis, and timely insulin secretion collectively maintain blood glucose concentrations within a stable range. In T2DM, impaired insulin signaling markedly reduces glucose uptake by peripheral tissues, whereas hepatic glucose production remains inappropriately elevated owing to impaired insulin-mediated suppression of gluconeogenesis (8). These abnormalities together constitute the pathophysiological basis of both fasting and postprandial hyperglycemia.

Recent advances in metabolomics have enabled comprehensive, high-throughput qualitative and quantitative profiling of small-molecule metabolites in biological samples, thereby providing a systematic characterization of metabolic alterations associated with T2DM (9). A large body of evidence consistently demonstrates that the metabolome of T2DM patients exhibits characteristic abnormalities in glucose metabolism and energy metabolism pathways, most prominently a compensatory upregulation of the glycolytic pathway coupled with restricted oxidative flux through the tricarboxylic acid (TCA) cycle.

#### 3.1.1 Glycolytic pathway

Within the glycolytic pathway, impaired glucose uptake and utilization by peripheral tissues result in persistent hyperglycemia and are accompanied by the accumulation of glycolytic intermediates, particularly pyruvate and lactate (10). Studies have shown that serum lactate levels are significantly increased in T2DM patients (11), reflecting both a compensatory enhancement of glycolytic flux and a reduction in mitochondrial oxidative capacity (12). The accumulation of pyruvate is associated with impaired mitochondrial oxidative phosphorylation, which restricts its entry into the TCA cycle.

#### 3.1.2 TCA cycle

In contrast to enhanced glycolysis, oxidative metabolism through the tricarboxylic acid (TCA) cycle is markedly impaired in T2DM (13). As the central oxidative pathway integrating carbohydrate, lipid, and amino acid metabolism, the TCA cycle plays a pivotal role in cellular energy production. This suppression is closely associated with mitochondrial dysfunction, a reduced NAD^+^/NADH ratio, and impaired conversion of pyruvate to acetyl-coenzyme A (acetyl-CoA) (14).

Metabolomics analyses have revealed that TCA cycle flux is inhibited in both skeletal muscle and the systemic circulation of patients with T2DM, accompanied by aberrant accumulation of lactate and pyruvate, the terminal products of glycolysis (13). These changes give rise to the metabolic signature of “enhanced glycolysis coupled with restricted oxidative capacity of the TCA cycle”. Such aberrant metabolic alterations are not merely a downstream consequence of disease onset and progression; they in turn further exacerbate IR and mitochondrial dysfunction, forming a self-perpetuating vicious cycle (14).

### 3.2 Amino acid metabolism

#### 3.2.1 Branched-chain amino acid metabolism

Branched-chain amino acids (BCAAs), comprising leucine, isoleucine, and valine, have been extensively validated by metabolomics research for their association with the risk of T2DM. Circulating BCAA concentrations are markedly elevated in patients with T2DM relative to healthy populations (15), and exhibit a significant positive correlation with glycemic traits (16).

An untargeted metabolomics analysis conducted by Menni et al. involving 2,204 British women identified a strong association between BCAAs and impaired fasting glucose (17). In a prospective analysis of the Framingham Heart Study, Wang et al. demonstrated that individuals with baseline BCAA levels in the highest quartile had a 2.0- to 3.5-fold greater risk of developing T2DM over 12 years of follow-up, compared with those in the lowest quartile (18). Among middle-aged and older populations with diabetes or hypertension, elevated BCAA levels are also found to be partially associated with increased mortality risk (19).

Abnormally elevated circulating BCAA levels mediate the pathogenesis of IR via multiple mechanistic pathways. BCAAs activate the mammalian target of rapamycin (mTOR) signaling cascade, downregulate the activity of insulin receptor substrate 1 (IRS-1) (20), and consequently impair insulin signal transduction. In addition, excessive BCAA accumulation drives incomplete fatty acid oxidation and compromises mitochondrial function, which further leads to impaired TCA cycle flux and the buildup of lipids and acylcarnitines, thereby exacerbating IR at the metabolic level (21). Furthermore, several metabolites generated during BCAA catabolism, including branched-chain α-keto acids (BCKAs), 3-hydroxyisobutyric acid (3-HIB), and β-aminoisobutyric acid (BAIBA), have emerged as important metabolic regulators. These metabolites directly or indirectly modulate insulin signaling and fatty acid oxidative metabolism, further linking impaired BCAA metabolism to metabolic dysfunction in T2DM (22).

#### 3.2.2 Aromatic amino acid metabolism

Aromatic amino acids (AAAs), primarily phenylalanine and tyrosine, are consistently associated with an increased risk of incident T2DM (23). Prospective cohort studies have demonstrated that elevated baseline plasma phenylalanine concentrations predict the future development of T2DM in normoglycemic individuals (18).

Mechanistically, altered AAA metabolism may reflect dysregulated hepatic amino acid metabolism and impaired metabolic flexibility, potentially contributing to the accumulation of phenylalanine and tyrosine observed in insulin-resistant states (14, 24). Accumulating evidence suggests that elevated circulating AAAs may reflect early metabolic inflexibility and impaired insulin sensitivity before the onset of overt diabetes (25). Furthermore, excessive accumulation of phenylalanine and tyrosine has been linked to chronic low-grade inflammation and oxidative stress, both of which contribute to the development of insulin resistance and pancreatic β-cell dysfunction (26). These findings indicate that altered AAA metabolism is not merely a metabolic consequence of T2DM but may also participate in disease progression.

#### 3.2.3 Metabolism of other amino acids

Among other amino acids, the association between glycine and diabetes exhibits prominent population-specific heterogeneity: an inverse association is predominantly observed in European populations (27), whereas a positive association has been detected in Chinese populations (28). Mendelian randomization analysis from the Framingham Heart Study revealed that genetically predicted glycine levels are inversely associated with diabetes risk (29), suggesting a potential protective role of glycine; however, this association is modulated by ethnic and dietary factors.

Alanine serves as a critical substrate for hepatic gluconeogenesis. Accumulating evidence confirms that baseline circulating alanine concentrations are significantly associated with elevated diabetes risk and IR (30). Following transamination, alanine is converted to pyruvate and glutamate, thereby participating in the dysregulation of glucose metabolism.

Glutamate and glutamine exert opposite effects on diabetes risk (31). Elevated plasma glutamate levels are positively correlated with an increased risk of incident T2DM, whereas glutamine levels show an inverse correlation with such risk (32). Mechanistically, glutamate may increase the susceptibility of pancreatic β-cells to oxidative damage by promoting oxidative stress, which subsequently leads to β-cell dysfunction. In contrast, glutamine exerts a protective effect by stimulating glucagon-like peptide-1 (GLP-1) secretion and reducing postprandial blood glucose levels.

However, the relationship between circulating amino acids and T2DM is not uniformly consistent across populations. Although elevated BCAAs and aromatic amino acids have been repeatedly associated with insulin resistance and future diabetes risk, the magnitude of these associations may vary across populations and metabolic contexts (18, 27, 28). Sex-specific differences have also been reported, suggesting that the metabolic significance of circulating BCAAs and AAAs may not be uniform across individuals (33). Moreover, population-specific heterogeneity has been observed for individual amino acids. For example, inverse associations between glycine and diabetes risk have been reported in European populations, whereas positive associations have been observed in Chinese populations (27, 28); conversely, Mendelian randomization evidence has suggested an inverse association between genetically predicted glycine levels and diabetes risk (29). In addition, circulating amino acid concentrations reflect the complex interplay between amino acid availability, tissue uptake, protein turnover, and metabolic utilization, rather than representing a direct readout of a single metabolic pathway (21). Therefore, BCAAs and AAAs should be interpreted as components of a broader metabolic signature rather than isolated biomarkers of T2DM.

### 3.3 Lipid metabolism

Lipids are essential components of cellular energy homeostasis and serve diverse biological functions, acting as metabolic substrates, signaling molecules, and structural constituents of cell membranes. Under physiological conditions, adipose tissue maintains relatively stable levels of free fatty acids (FFAs) by regulating the balance between lipolysis and lipid storage.

Dysregulated lipid metabolism, particularly elevated triglyceride levels, has been well documented in clinical studies as a classical risk factor for the development of T2DM (34). In the setting of T2DM, the antilipolytic effect of insulin on adipose tissue is markedly attenuated (32), resulting in excessive activation of adipose tissue lipolysis and aberrantly elevated circulating FFA concentrations. Excess FFAs overflow into peripheral tissues such as the liver and skeletal muscle, exceeding the oxidative metabolic capacity of mitochondria. On one hand, this surplus triggers compensatory dysregulation of fatty acid oxidative pathways and disrupts the normal functioning of glucose metabolic pathways. On the other hand, it drives the ectopic intracellular deposition of abundant lipotoxic intermediate metabolites, including diacylglycerol, ceramide, and acylcarnitine, which further exacerbates IR and mitochondrial functional impairment (35).

#### 3.3.1 Fatty acid oxidation

As early as the 1960s, Randle et al. proposed the competitive relationship between fatty acids and glucose during oxidative metabolism, and demonstrated that excessive fatty acid oxidation impairs glucose homeostasis and induces IR (36). Furthermore, the intracellular accumulation of lipotoxic lipid intermediates, including diacylglycerol, triacylglycerol, and ceramide, is also closely associated with IR.In the insulin-sensitive state, insulin regulates serum glycerol and FFA levels by inhibiting lipolysis. Conversely, in the setting of IR, enhanced lipolysis drives excessive hydrolysis of triglycerides, leading to the massive production of glycerol and FFAs. Findings from the METSIM study further indicate that saturated fatty acid levels are positively associated with diabetes risk, whereas unsaturated fatty acids exhibit an inverse association (37).

#### 3.3.2 Acylcarnitine accumulation

Acylcarnitines are key intermediate metabolites generated during fatty acid β-oxidation, and their production and accumulation directly reflect the efficiency of mitochondrial fatty acid oxidation (38). Animal model studies have demonstrated that acylcarnitine accumulation is associated with β-oxidation dysfunction, mitochondrial stress, and IR (39). The pathophysiological roles of these metabolites appear to depend on acyl chain length: short-chain acylcarnitines are linked to BCAA catabolism, whereas long-chain species may reflect incomplete fatty acid oxidation (40).

## 4 Core mechanisms underlying exercise intervention-mediated amelioration of metabolic dysregulation in T2DM

As a core non-pharmacological strategy for the prevention and management of T2DM, regular exercise systematically reverses the markedly dysregulated state of key metabolites and metabolic pathways under pathological conditions via multiple mechanisms, including skeletal muscle contraction, the release of endocrine signals, and the remodeling of mitochondrial function (**Table 1**).

**Table 1:** List of metabolomics studies related to exercise intervention in type 2 diabetes.

Study	Subjects	Exercise intervention	Metabolomics platform	Major metabolic alterations
Human studies				
Contrepois et al. (41) (2020)	36 insulin-resistant adults (40–75 years)	Acute symptom-limited exercise test	LC–MS	Acute exercise induced dynamic changes in glycolytic metabolites (lactate and pyruvate) and tricarboxylic acid (TCA) cycle intermediates
Lee et al. (42) (2021)	26 sedentary middle-aged men	Combined endurance and resistance training for 12 weeks	GC–MS/MS; HPLC–MS/MS	Long-term exercise markedly enhanced branched-chain amino acid (BCAA) catabolism in skeletal muscle and subcutaneous white adipose tissue.
Nayor et al. (43) (2021)	471 middle-aged and older adults	Acute cardiopulmonary exercise test	LC–MS	A total of 502 plasma metabolites, including lactate, pyruvate, and succinate, changed significantly immediately after exercise.
Savikj et al. (44) (2022)	8 men with type 2 diabetes mellitus	High-intensity interval training (HIIT) for 2 weeks	UPLC–MS	HIIT increased plasma diacylglycerols, skeletal muscle acylcarnitines, and sphingolipids and lysophospholipids in subcutaneous adipose tissue following both morning and afternoon exercise sessions.
Zhang et al. (45) (2024)	20 patients with type 2 diabetes mellitus	Tai Chi or brisk walking for 12 weeks	LC–MS	Tai Chi may improve impaired BCAA metabolism, whereas brisk walking primarily regulates steroid hormone biosynthesis and arachidonic acid metabolism.
Duft et al. (46) (2024)	131 sedentary adults	Combined aerobic and resistance training for 16 weeks	UPLC–MS	Combined exercise improved lipid metabolic profiles by reducing saturated and monounsaturated lipid species while increasing polyunsaturated lipid species.
Animal studies				
Li et al. (47) (2016)	Sprague Dawley rats	Swimming training for 8 weeks	UPLC–QTOF–MS	Swimming training restored polyunsaturated fatty acids and altered arginine and carnitine metabolism.
Xiang et al. (48) (2018)	db/db mice	Moderate-intensity treadmill running for 5 weeks	LC–MS; GC–MS	Exercise reduced long-chain acylcarnitines and long-chain fatty acids in skeletal muscle and restored lactate levels to near-normal values.
Khoramipour et al. (49) (2024)	Sprague Dawley rats	High-intensity interval training for 8 weeks	NMR	HIIT improved amino acid metabolism, with significant alterations in valine, leucine, isoleucine, alanine, and other amino acid metabolites.
Wang et al. (50) (2024)	SD rats	Treadmill exercise for 8 weeks	UPLC-QTOF-MS	Exercise ameliorated diabetic myocardial metabolic dysfunction by promoting fatty acid oxidation and regulating TCA cycle- and antioxidant-related metabolic pathways.

LC–MS, liquid chromatography–mass spectrometry; UPLC, ultra-performance liquid chromatography; QTOF, quadrupole time-of-flight; UPLC–QTOF–MS, ultra-performance liquid chromatography–quadrupole time-of-flight–mass spectrometry; GC–MS/MS, gas chromatography–tandem mass spectrometry; HPLC–MS/MS, high-performance liquid chromatography–tandem mass spectrometry; NMR, nuclear magnetic resonance; HIIT, high-intensity interval training; TCA, tricarboxylic acid cycle.

### 4.1 Restoring TCA cycle flux and alleviating glycolytic stress

Metabolomics analyses have revealed that patients with T2DM exhibit the metabolic signature of “enhanced glycolysis coupled with restricted oxidative capacity of the TCA cycle”, and exercise intervention can effectively reverse this metabolic dysregulation. By activating the AMP-activated protein kinase/peroxisome proliferator-activated receptor gamma coactivator 1-alpha (AMPK-PGC-1α) signaling axis, exercise redirects glycolytic products in patients with T2DM back into the TCA cycle for oxidative metabolism, leading to reduced levels of accumulated metabolites such as lactate and pyruvate. Meanwhile, levels of TCA cycle intermediates in skeletal muscle and plasma are elevated, reversing the core defect of suppressed TCA cycle activity under diabetic conditions.

#### 4.1.1 Acute exercise

Using multi-omics time-series analysis, Contrepois et al. captured rapid elevations in plasma lactate and pyruvate within 2 minutes after acute exercise, which occurred in parallel with increases in TCA cycle intermediates (malate, citrate, and α-ketoglutarate) and were accompanied by a sharp rise in markers of ATP turnover (hypoxanthine and xanthine) (41). This kinetic profile indicates that exercise re-establishes the metabolic coupling of glycolytic products to the TCA cycle, rapidly channeling previously accumulated glycolytic intermediates into mitochondria for oxidative phosphorylation.

Quantitative metabolomic analyses by Nayor and Shah et al. demonstrated that acute exercise increased plasma lactate and pyruvate levels by 246% and 75%, respectively. Simultaneously, the TCA cycle intermediates fumarate and succinate, together with pantothenate, were significantly elevated. In contrast, the glycolytic intermediate 3-phosphoglycerate decreased by 31% (43), indicating that enhanced TCA cycle flux promoted the oxidative utilization of glycolytic intermediates, thereby relieving glycolytic stress and improving metabolic efficiency.

#### 4.1.2 Long-term regular exercise

Notably, the effects of exercise training on metabolites such as lactate exhibit time-dependent differences: acute exercise causes a transient elevation in lactate, whereas long-term regular training helps reduce lactate accumulation in the resting state. Savikj et al., in a randomized crossover trial conducted in T2DM patients, revealed the long-term metabolic adaptation characteristics induced by high-intensity interval training. After completing a two-week program comprising six sessions of high-intensity interval training, the TCA cycle pathway in skeletal muscle was significantly activated, with multiple key metabolites showing coordinated increases, while glycolytic pathway metabolites in subcutaneous adipose tissue were consistently downregulated (44). This finding indicates that exercise training not only elicits acute metabolic flux changes but also induces sustained metabolic pattern remodeling, shifting the metabolic profile of T2DM patients from inefficient glycolysis toward oxidative metabolism dominance, with elevated resting-state TCA cycle flux and reduced long-term reliance on the glycolytic system.

### 4.2 Accelerating amino acid catabolism and ameliorating dysregulated amino acid metabolism

Exercise exerts systemic modulatory effects on the amino acid metabolic dysregulation associated with T2DM. Quantitative metabolomic analyses demonstrate that exercise upregulates the activity of the branched-chain α-keto acid dehydrogenase complex (BCKDC) in skeletal muscle, accelerates the oxidative catabolism of leucine, isoleucine, and valine, and markedly reduces the abnormally elevated circulating BCAA levels in patients with T2DM.

With respect to AAAs, regular exercise likewise ameliorates the aberrant accumulation of phenylalanine and tyrosine, facilitating the correction of their metabolic dysregulation in the diabetic state. Concurrently, exercise increases plasma glycine concentrations while lowering glutamate levels, thereby normalizing the aberrantly elevated glutamate-to-glutamine ratio. These metabolic changes contribute to mitigating the risk of oxidative damage to pancreatic β-cells and improving insulin secretory function.

#### 4.2.1 Combined training

Lee et al. demonstrated that 12 weeks of combined endurance and resistance training in men with dysglycemia and normoglycemic controls significantly upregulated BCKDC gene expression in both skeletal muscle and adipose tissue, enhancing the mitochondrial oxidative catabolic capacity of BCAAs (42). Concurrently, exercise was found to ameliorate the aberrant accumulation of AAAs (phenylalanine and tyrosine), and reverse the metabolic buildup driven by shared transporters for BCAAs and AAAs.

#### 4.2.2 High-intensity interval training

Using metabolomics approaches, Khoramipour et al. revealed that multiple amino acid metabolic pathways were markedly dysregulated in the skeletal muscle of rats following diabetes induction, and 8 weeks of high-intensity interval training (HIIT) effectively reversed these abnormalities.With regard to BCAA metabolism, exercise significantly modulates the biosynthetic pathways of valine, leucine, and isoleucine. By enhancing the oxidative utilization of these amino acids in skeletal muscle, exercise channels accumulated BCAA pools into the TCA cycle for energy production.In terms of alanine, aspartate, and glutamate metabolism, exercise strengthens the glucose-alanine cycle in skeletal muscle and its metabolic coupling with the liver. This promotes amino nitrogen transport and the efflux of gluconeogenic precursors, thereby alleviating metabolic inflexibility under diabetic conditions.The synergistic remodeling of these amino acid metabolic pathways collectively constitutes the metabolic basis underlying HIIT-induced reductions in fasting blood glucose and IR index in diabetic rats (49).

### 4.3 Promoting fatty acid oxidation and clearing lipotoxic intermediate metabolites

T2DM is characterized by profound lipid metabolic dysregulation, including elevated circulating FFAs, accumulation of long-chain acylcarnitines, increased ceramide production, and ectopic lipid deposition. These lipotoxic alterations contribute to impaired insulin signaling, mitochondrial dysfunction, and chronic low-grade inflammation. Exercise intervention effectively restores lipid metabolic homeostasis by enhancing mitochondrial fatty acid oxidation, reducing lipotoxic metabolite accumulation, and improving metabolic flexibility.

Mechanistically, these adaptations are closely associated with activation of the PPARα–PGC-1α signaling pathway, which promotes mitochondrial biogenesis, fatty acid transport, and β-oxidation while suppressing the accumulation of lipotoxic intermediates (51, 52). Consequently, exercise enhances metabolic flexibility and restores efficient substrate switching between lipid and glucose oxidation.

#### 4.3.1 Combined training

In a randomized controlled trial (RCT) conducted by Duft et al. in middle-aged patients with T2DM, 16 weeks of moderate-intensity combined training reduced fat mass and waist circumference, increased fat-free mass, significantly improved cardiorespiratory fitness, and alleviated IR.At the level of lipid metabolism, exercise effectively lowered plasma and white adipose tissue (WAT) levels of sphingolipids (e.g., ceramides, sphingomyelins, hexosylceramides), glycerolipids (e.g., diacylglycerols, triacylglycerols), and glycerophospholipids (e.g., phosphatidylcholine, phosphatidylethanolamine, phosphatidic acid) enriched with saturated and monounsaturated fatty acid chains (46). All of these lipid species are closely implicated in IR, lipotoxicity, and mitochondrial dysfunction.In a T2DM rat model, Li and Liu demonstrated that 8 weeks of swimming training markedly restored serum levels of polyunsaturated fatty acids including eicosapentaenoic acid (EPA) and linolenic acid, while concurrently reducing arginine and carnitine concentrations, thereby restoring these metabolites to levels nearly comparable to those of normal control animals (47).

#### 4.3.2 High-intensity interval training

Using multi-tissue metabolomics profiling, Savikj et al. revealed that acylcarnitines serve as a central node in exercise-regulated cross-tissue metabolic crosstalk. Regardless of training duration, acylcarnitines in skeletal muscle, adipose tissue, and plasma exhibit coordinated alterations and form a tight regulatory network with BCAA, fatty acid, and glutathione metabolites, which mechanistically links exercise-enhanced TCA cycle flux to systemic lipid mobilization and antioxidant defense.The study further demonstrated that afternoon training induces greater increases in skeletal muscle lipid content and mitochondrial complex III abundance compared with morning training, underscoring a distinct diurnal divergence where exercise timing interacts with peripheral circadian clocks to dictate oxidative metabolic adaptation in the diabetic phenotype (44).

**Figure 1:**
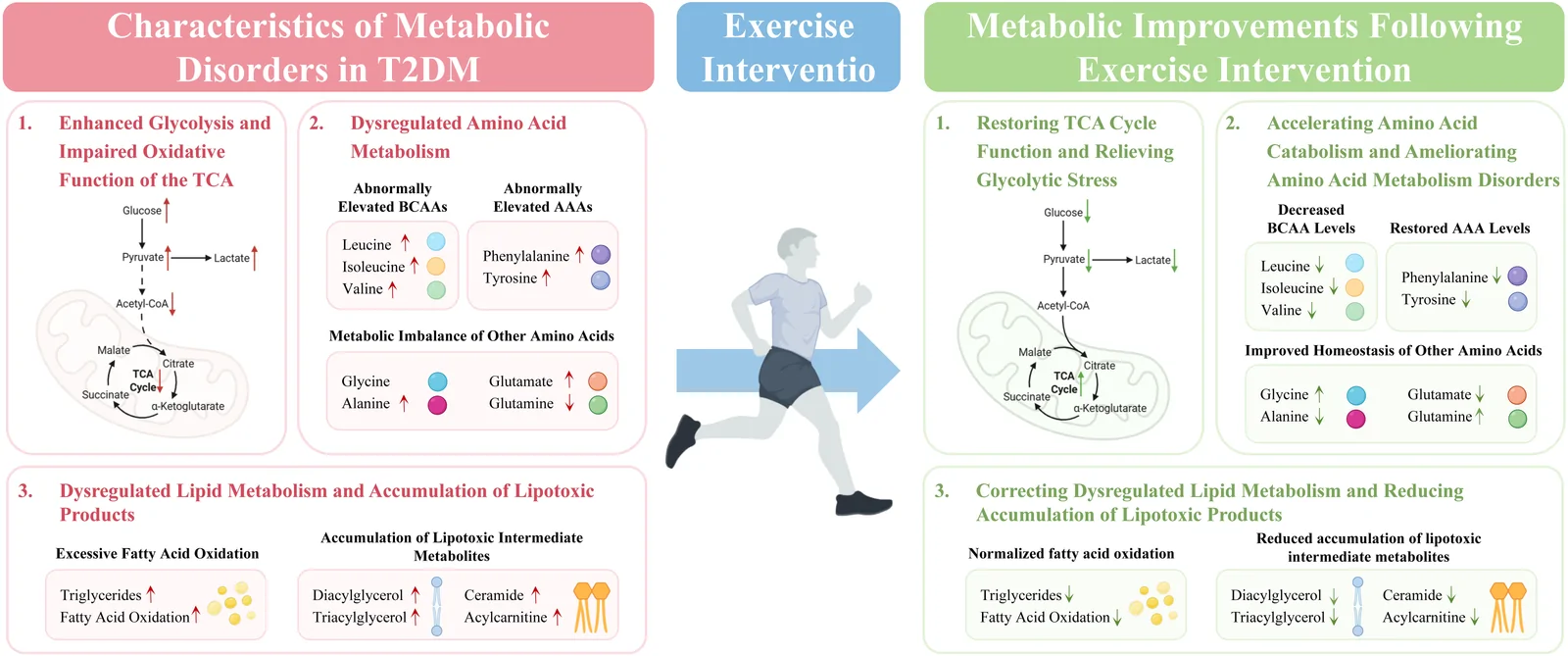
Metabolic disturbances in T2DM and the metabolic improvements induced by exercise intervention. T2DM, type 2 diabetes mellitus; TCA, tricarboxylic acid; BCAA, branched-chain amino acid; AAA, aromatic amino acid.

Collectively, exercise training systematically restores glucolipid metabolic homeostasis in patients with T2DM by remodeling lipid metabolic profiles, reducing the accumulation of lipotoxic intermediate metabolites, and enhancing mitochondrial oxidative capacity (**Figure 1**).

An important limitation of the current evidence is the lack of a clearly established dose-response relationship between exercise and metabolomic remodeling in T2DM. Acute exercise and chronic training may induce distinct metabolic responses, reflecting differences in the temporal dynamics of exercise-induced metabolic remodeling, while exercise intensity, duration, frequency, and modality can substantially influence substrate utilization and metabolite trajectories (**Table 1**). For example, acute exercise can induce pronounced changes in glycolytic and tricarboxylic acid cycle-related metabolites, whereas longer-term moderate-intensity or combined training may promote more sustained adaptations in amino acid and lipid metabolism (41, 42, 46). Moreno-Cabañas et al. further demonstrated that different exercise interventions can produce distinct metabolic flux responses in individuals with T2DM, underscoring the importance of exercise intensity and modality when interpreting metabolomic alterations (53). Therefore, metabolomic responses should not be interpreted independently of exercise dose. At present, the available evidence does not support a universal metabolite-defined exercise dose for T2DM, highlighting the need for dose-ranging, longitudinal, and adequately powered intervention studies.

## 5 Overview of metabolomics

The metabolic adaptations described above provide a mechanistic basis for understanding how exercise ameliorates metabolic dysfunction in T2DM. However, the clinical value of these metabolic changes extends beyond mechanistic interpretation. Because individuals with T2DM may exhibit heterogeneous responses to the same exercise intervention, metabolomics provides an opportunity to translate metabolic signatures into clinically relevant biomarkers. Baseline metabolic profiles may help identify individuals who are more likely to respond favorably to specific exercise regimens, whereas longitudinal changes in metabolites may provide complementary information for monitoring intervention-related metabolic adaptations. Therefore, metabolomics may serve as a bridge between mechanistic characterization and precision exercise prescription by facilitating the prediction, monitoring, and adjustment of exercise interventions.

**Figure 2:**
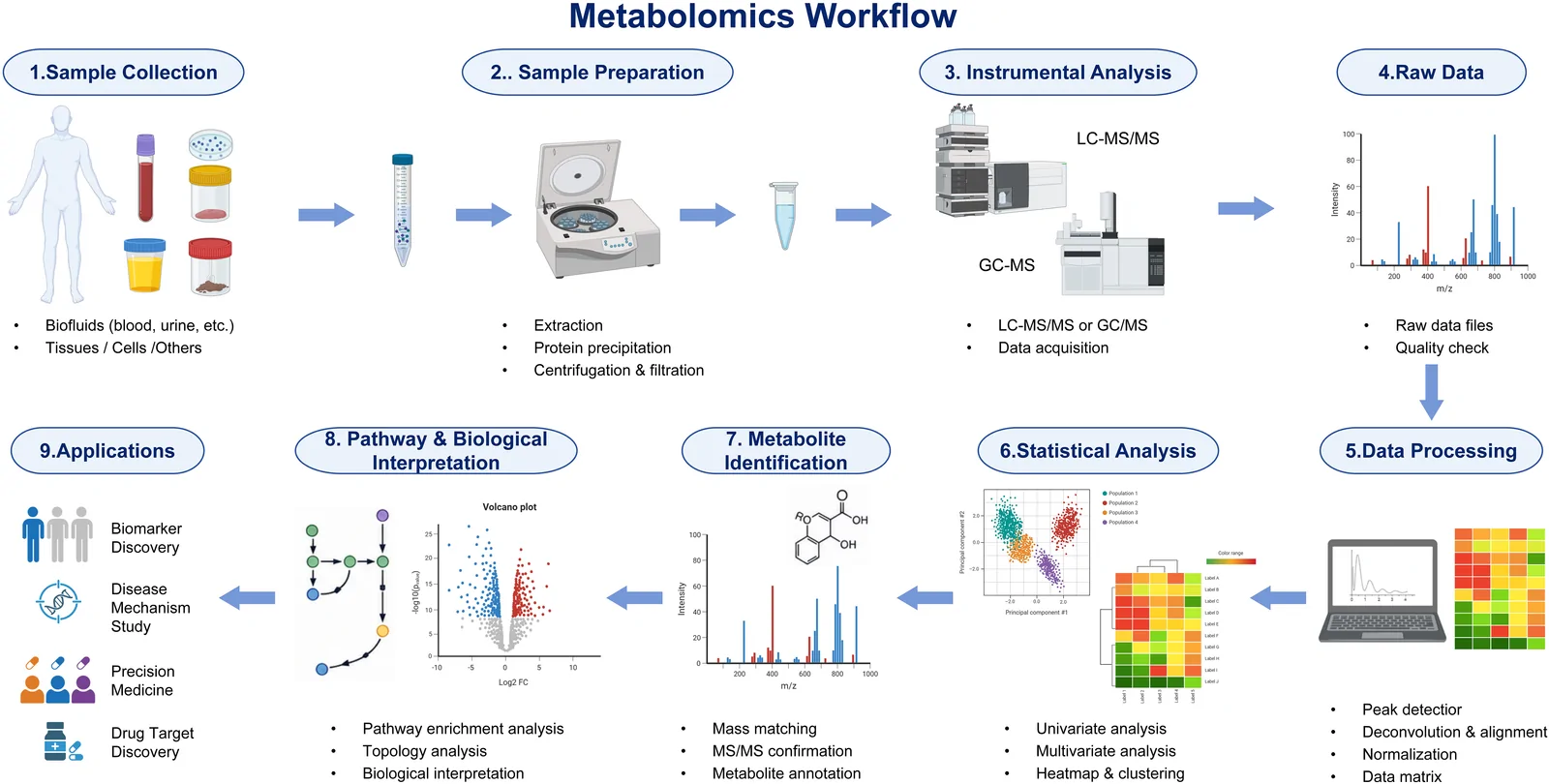
General workflow of metabolomics analysis.

Following genomics, transcriptomics, and proteomics, metabolomics is the branch of systems biology positioned closest to the downstream end products of biological processes (54). It aims to conduct qualitative and quantitative profiling of all low-molecular-weight metabolites in living organisms and characterize their dynamic alteration patterns, thereby elucidating the intrinsic associations between metabolites and physiological/pathological processes, environmental factors, and genetic variations (**Figure 2**) (55, 56).

With the continuous advancement of analytical technologies including mass spectrometry (MS) and nuclear magnetic resonance (NMR), metabolomics has evolved into a diversified technological framework encompassing untargeted, targeted, pseudo-targeted approaches, and metabolic fluxomics. These methodologies enable a broad spectrum of research applications, ranging from global metabolite screening to precise quantification, and from static compositional detection to dynamic flux analysis. In the field of T2DM research, metabolomics applications span multiple core domains: early risk prediction, pathogenesis elucidation, and biomarker screening for complications (9, 57, 58). Meanwhile, it provides critical technical support for mechanistic investigation, efficacy evaluation, and precise clinical translation of non-pharmacological therapeutic strategies represented by exercise intervention (5, 52, 59).

### 5.1 Classification of metabolomics technologies

#### 5.1.1 Untargeted metabolomics

Untargeted metabolomics, also referred to as discovery metabolomics, aims to characterize the composition and dynamic alteration patterns of metabolites in biological samples with the broadest possible coverage (55). This approach typically employs high-resolution mass spectrometry or NMR spectroscopy, enabling simultaneous detection of hundreds to thousands of metabolites. With its extensive coverage, it is particularly suited for identifying novel metabolic biomarkers and dysregulated pathways (60).

In research on exercise intervention for T2DM, untargeted metabolomics is widely utilized to explore the underlying mechanisms through which exercise ameliorates metabolic dysregulation. Accumulating studies have demonstrated that long-term exercise significantly modulates multiple metabolic pathways, including lipid metabolism, the TCA cycle, amino acid metabolism, and bile acid metabolism, and drives the remodeling of systemic energy metabolism (5, 43).Nevertheless, owing to the complexity of raw detection datasets, the technical challenges associated with metabolite identification and data analysis, and the predominantly relative quantitative nature of its outputs, findings from untargeted metabolomics generally require subsequent validation via targeted metabolomics (61).

#### 5.1.2 Targeted metabolomics

Building on untargeted metabolomics, targeted metabolomics performs qualitative and quantitative analysis of known metabolites and enables precise detection of key metabolites within specific metabolic pathways (61). Using targeted quantitative mass spectrometry, Møller et al. demonstrated that insulin-mediated clearance of plasma BCAAs is markedly impaired in patients with T2DM, whereas acute exercise reduces circulating BCAA levels via insulin-independent pathways, revealing an independent regulatory mechanism by which exercise ameliorates amino acid metabolic dysregulation (62).

This approach features high sensitivity, accurate quantification, and excellent reproducibility, and yields absolute quantitative data for target metabolites. It is suitable for in-depth dissection of specific metabolic pathways and validation of therapeutic efficacy biomarkers (63).

#### 5.1.3 Pseudo-targeted metabolomics

Pseudo-targeted metabolomics is a novel metabolomic analytical strategy that integrates the strengths of both non-targeted and targeted approaches. In this workflow, high-resolution mass spectrometry is first employed to perform non-targeted global profiling of samples, acquiring mass spectral information on all detectable metabolites and screening for characteristic ion pairs. Based on these ion pairs, a targeted-like multiple reaction monitoring (MRM) detection method is subsequently established, ultimately enabling accurate quantification of a large number of metabolites (64). This technique retains the broad coverage characteristic of non-targeted approaches while offering the high sensitivity and quantitative accuracy of targeted methods (65). However, it is highly dependent on the quality of data extraction from the initial non-targeted analysis, and the preparatory phase is relatively time-consuming.

#### 5.1.4 Metabolic fluxomics

Metabolic fluxomics employs isotope tracing technologies (e.g., carbon-13 [¹³C] tracers) to dynamically monitor the trafficking of labeled atoms across metabolic pathways and quantify the flux of individual metabolic reactions (66). By transcending the inherent constraints of static concentration measurements, this approach provides dynamic flux insights into metabolic networks. Nevertheless, it requires sophisticated experimental design and operational procedures, rendering it unsuitable for large-scale screening campaigns. It is particularly well suited for examining energy transfer in core metabolic organs during exercise and for in-depth dissection of mitochondrial function.

Using stable isotope tracing-coupled metabolic flux analysis, Moreno-Cabañas et al. demonstrated that the combination of metformin and HIIT synergistically ameliorates postprandial insulin sensitivity in patients with T2DM. This beneficial effect is achieved by targeting key metabolic nodes, namely suppressing hepatic endogenous glucose production and enhancing peripheral glucose utilization, thereby providing flux-level evidence for exercise-pharmacological combination therapy in diabetes management (53).

**Figure 3:**
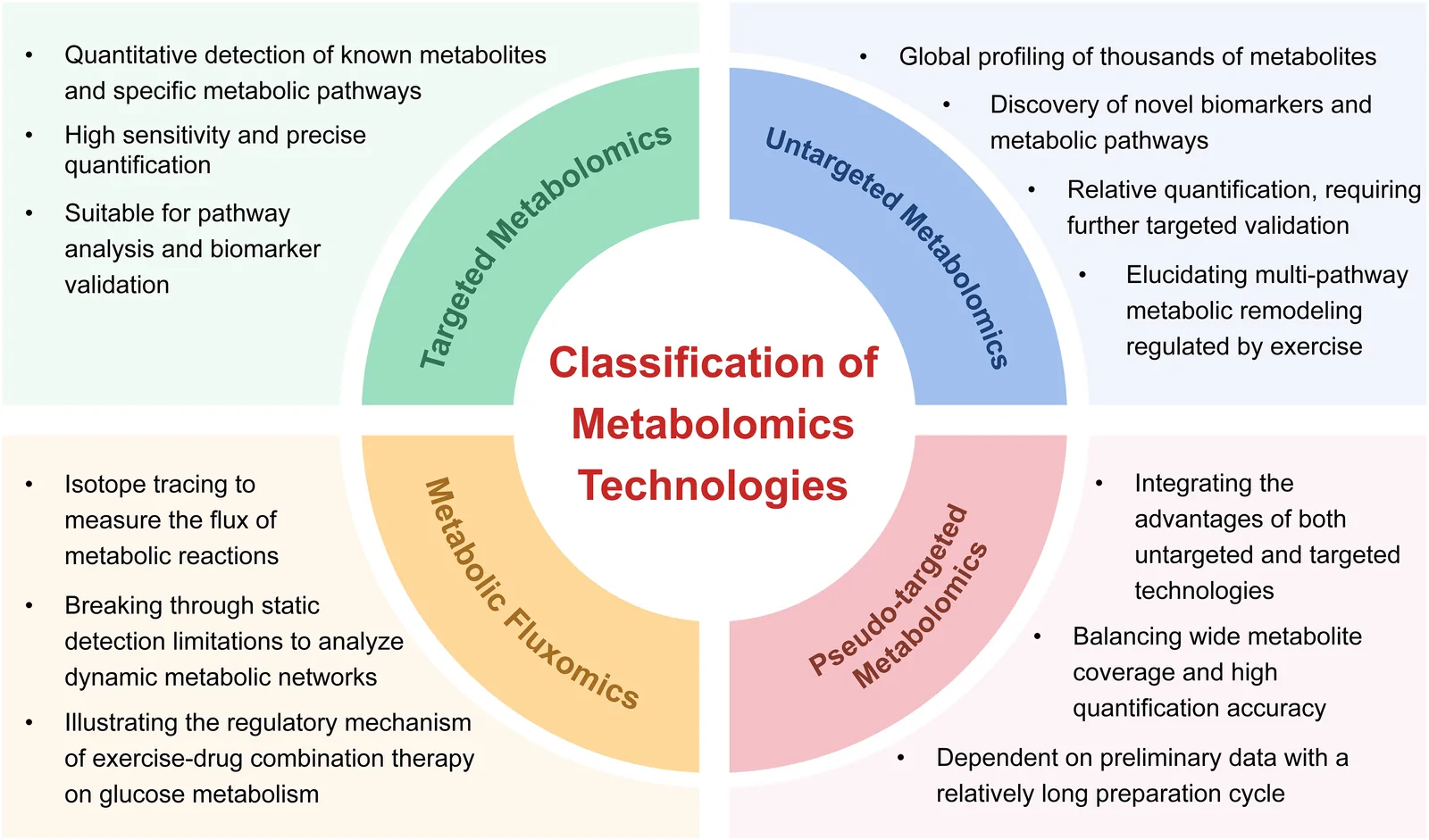
Classification and core characteristics of metabolomics technologies.

Collectively, these metabolomic approaches are complementary rather than competitive (67). Untargeted metabolomics is primarily used for biomarker discovery and hypothesis generation; targeted metabolomics provides accurate quantitative validation; pseudo-targeted metabolomics balances analytical coverage and quantitative performance; whereas metabolic fluxomics uniquely characterizes dynamic metabolic activity. Integrating these complementary techniques enables comprehensive characterization of exercise-induced metabolic remodeling and provides robust evidence for precision exercise interventions in T2DM (**Figure 3**).

### 5.2 Applications of metabolomics technologies in T2DM research

#### 5.2.1 Elucidation of pathogenesis

Metabolomics facilitates the identification of metabolic aberrations linked to IR and pancreatic β-cell dysfunction. Accumulating studies have demonstrated that circulating levels of multiple metabolites, including BCAAs (16), AAAs (18), FFAs (68), and acylcarnitines (69), are markedly elevated in patients with T2DM. These metabolic dysregulations are closely associated with impaired insulin signaling, mitochondrial dysfunction, and chronic inflammation.By characterizing the alterations of these metabolites, the molecular mechanisms underlying the onset and progression of T2DM can be elucidated from a systems biology perspective, providing a theoretical foundation for the prevention and management of the disease (4).

#### 5.2.2 Early diagnosis and risk prediction

With the continuous advancement of metabolomics and data analysis technologies, accumulating studies have demonstrated that select metabolites can identify metabolic aberrations prior to the onset of clinical symptoms. For instance, alterations in plasma levels of BCAAs, AAAs, phospholipids, and fatty acid metabolites can predict the risk of incident T2DM in advance (31, 68). Metabolomics holds great promise for developing more sensitive predictive models, enabling early screening and precision prevention of diabetes mellitus (16, 68, 70, 71).

#### 5.2.3 Biomarker screening for diabetic complications

The onset and progression of diabetic kidney disease (DKD), diabetic retinopathy, and cardiovascular complications are all accompanied by specific metabolic alterations (58, 72, 73). By detecting changes in metabolic profiles in blood, urine, and tissues, researchers can identify key metabolites associated with these complications. For instance, patients with DKD exhibit marked lipid metabolic dysregulation, manifested as altered levels of lipid species including phosphatidylcholines and sphingomyelins, and these changes are closely associated with the severity of renal function impairment (74, 75). Such metabolic signatures not only contribute to elucidating the pathogenesis of diabetic complications, but also provide novel biomarkers for disease monitoring and prognostic evaluation.

### 5.3 Clinical translational applications of metabolomics in exercise intervention for T2DM

#### 5.3.1 Prediction of exercise therapeutic efficacy

Recent studies have demonstrated that baseline levels of BCAAs (42), specific ceramide species (76), and soluble interleukin-6 receptor (sIL-6R) (77) can effectively predict individual glycemic improvement in response to exercise intervention.

Recent evidence suggests that circulating biomarkers may help stratify individuals according to their exercise responsiveness. For example, baseline soluble interleukin-6 receptor (sIL-6R) levels have been associated with differential glycemic responses to exercise (77). However, this finding should currently be considered evidence for response stratification rather than a validated metabolomics-guided exercise prescription strategy, as it remains unclear whether individuals with different sIL-6R profiles would benefit from different exercise modalities, intensities, or durations. Larger, prospective, multicenter studies are needed to validate these predictive signatures and determine their clinical utility.

#### 5.3.2 Monitoring of exercise adherence and dynamic evaluation of therapeutic efficacy

Regular exercise induces systemic metabolite alterations, including remodeling of the circulating acylcarnitine profile and fluctuations in TCA cycle intermediates (78, 79). Periodic measurement of these metabolites enables objective assessment of patients’ adherence to prescribed exercise regimens, thereby addressing the limitation of high subjectivity inherent in self-reported exercise logs (80).

Compared with glycated hemoglobin (HbA1c), which reflects average blood glucose levels over a 2 to 3 month period, changes in specific metabolites such as lactate, ketone bodies, and FFAs can capture the immediate effects of exercise intervention at an earlier stage (81, 82). Continuous tracking of metabolite dynamics also allows timely identification of suboptimal intervention efficacy or the development of therapeutic tolerance, providing evidence for the dynamic adjustment of exercise prescriptions.

#### 5.3.3 Exercise safety assessment: metabolic early warning indicators

Patients with diabetes mellitus commonly present with complications such as cardiovascular autonomic neuropathy and diabetic retinopathy, and therefore face substantially elevated exercise-related risks relative to healthy individuals (83, 84). Metabolomics provides an adjunctive approach to identify these associated risks.

Shared metabolic aberrations, including reduced concentrations of glycine and serine and dysregulated lipid profiles, have been identified in both T2DM and cardiovascular disease (85). Elevated plasma glutamate levels also serve as a common early predictive biomarker for both conditions.Ottosson et al. followed 1049 older adults free of metabolic disorders for 6.1 years and found that elevated baseline plasma glutamate levels were strongly associated with the incidence of both T2DM and coronary artery disease (86). These shared metabolic signatures indicate the presence of overlapping pathogenic pathways, suggesting that regular exercise training may exert concurrent beneficial effects on both disease entities.

## 6 Summary and perspectives

Exercise intervention represents a safe and effective non-pharmacological strategy for the prevention and management of T2DM. By restoring TCA cycle flux, accelerating the oxidative catabolism of BCAAs, and promoting fatty acid oxidation along with the clearance of lipotoxic metabolites, exercise systematically reverses metabolic dysregulations of glucose, amino acids, and lipids. Distinct exercise modalities and intervention timings have been shown to exert differential regulatory effects on these metabolic pathways. Future research should prioritize large-scale clinical intervention trials with long-term follow-up. Integrating individual baseline metabolic characteristics, studies should explore the optimal combination of exercise type, intensity, timing, and frequency to advance the establishment of precise, personalized exercise prescriptions. Meanwhile, further mechanistic elucidation of exercise responsiveness is warranted to improve the efficacy and safety of exercise-based therapeutics.

Metabolomics technologies provide critical methodological support for the elucidation of exercise mechanisms covered in this review. The complementary technological framework, consisting of untargeted, targeted, pseudo-targeted metabolomics and metabolic fluxomics, serves distinct research purposes respectively: global metabolite screening, precise validation, large-sample quantification, and dynamic flux analysis. This suite of approaches not only delineates the metabolic dysregulation profiles of T2DM and the regulatory mechanisms of exercise intervention, but also expands its translational value in therapeutic efficacy prediction, dynamic monitoring, and safety assessment.Nevertheless, several limitations remain in current research: most studies rely on single-omics analysis, detection standards lack uniformity, and clinical validation of candidate biomarkers is insufficient. Future efforts should prioritize the advancement of integrated multi-omics research, the establishment of standardized technical specifications, and the acceleration of clinical translation for metabolic biomarkers. These developments will provide a more solid theoretical foundation and practical guidance for the precise prevention and control of T2DM and the individualized implementation of exercise interventions.

From a clinical perspective, metabolomic profiles may complement conventional indicators such as HbA1c, glucose levels, body composition, and cardiorespiratory fitness to identify metabolic phenotypes, monitor early responses to exercise, and support individualized adjustment of exercise type and intensity. However, metabolomics should currently be considered an adjunctive rather than a standalone tool for exercise prescription, as predictive biomarkers and metabolic signatures require further validation. For researchers, future studies should incorporate repeated metabolomic measurements with standardized exercise protocols and clinically relevant outcomes to determine whether metabolic signatures can reliably predict exercise responsiveness and guide subsequent modifications of exercise interventions. These efforts will help translate metabolic insights into practical strategies for the precise prevention and management of T2DM and the individualized implementation of exercise interventions.
